# A Time-Driven Cloudlet Placement Strategy for Workflow Applications in Wireless Metropolitan Area Networks

**DOI:** 10.3390/s22093422

**Published:** 2022-04-29

**Authors:** Jianshan Zhang, Ming Li, Xianghan Zheng, Ching-Hsien Hsu

**Affiliations:** 1 College of Computer and Data Science, Fuzhou University, Fuzhou 350108, China; zhangjs0512@163.com (J.Z.); N190327047@fzu.edu.cn (M.L.); 2 Fujian Provincial Key Laboratory of Network Computing and Intelligent Information Processing, Fuzhou University, Fuzhou 350108, China; 3 Department of Computer Science and Information Engineering, Asia University, Taichung 413, Taiwan; robertchh@gmail.com

**Keywords:** mobile edge computing, wireless metropolitan area network, workflow application, cloudlet placement, computation offloading

## Abstract

With the rapid development of mobile technology, mobile applications have increasing requirements for computational resources, and mobile devices can no longer meet these requirements. Mobile edge computing (MEC) has emerged in this context and has brought innovation into the working mode of traditional cloud computing. By provisioning edge server placement, the computing power of the cloud center is distributed to the edge of the network. The abundant computational resources of edge servers compensate for the lack of mobile devices and shorten the communication delay between servers and users. Constituting a specific form of edge servers, cloudlets have been widely studied within academia and industry in recent years. However, existing studies have mainly focused on computation offloading for general computing tasks under fixed cloudlet placement positions. They ignored the impact on computation offloading results from cloudlet placement positions and data dependencies among mobile application components. In this paper, we study the cloudlet placement problem based on workflow applications (WAs) in wireless metropolitan area networks (WMANs). We devise a cloudlet placement strategy based on a particle swarm optimization algorithm using genetic algorithm operators with the encoding library updating mode (PGEL), which enables the cloudlet to be placed in appropriate positions. The simulation results show that the proposed strategy can obtain a near-optimal cloudlet placement scheme. Compared with other classic algorithms, this algorithm can reduce the execution time of WAs by 15.04–44.99%.

## 1. Introduction

With the emergence of more advanced mobile hardware and a new generation of mobile communication technology that integrates wireless communication and modern Internet technologies, many delay-sensitive and computation-intensive mobile applications, such as voice recognition applications, online games, webcasts, and augmented reality applications, are present on mobile devices. These applications pose new challenges to mobile devices. Although mobile-hardware-related technologies have made great progress, due to the restrictions of mobile devices in size, weight, battery life, and heat dissipation, the gap between the capability of limited computational resources and computation-intensive application requirements is gradually increasing [1]. Mobile cloud computing (MCC), a computing paradigm, integrates cloud computing and mobile computing to enhance the computation performance of mobile devices [2]. By offloading a portion or all of the components of mobile applications to a cloud server, the performance of mobile applications can be significantly improved [3]. However, modern mobile applications generally have high requirements for instantaneity. The traditional central cloud is usually remotely located and distant from the users. For most modern mobile applications, offloading to the central cloud may not be optimal. To overcome these disadvantages, mobile edge computing (MEC) is an efficient solution that can better solve the insufficient resources of mobile devices. As a new computing paradigm proposed after MCC, MEC has brought innovation to the working mode of traditional cloud computing. When edge servers are placed at the edge of the network, the computing power of the central cloud is distributed to the network edge, and the rich computational resources of the edge servers compensate for the lack of mobile devices. In particular, cloudlets have been widely studied by academia and industry in recent years, constituting a specific form of the edge server. The problem of cloudlet placement is the key to the efficient utilization of edge resources in the network [4,5,6]. A cloudlet is composed of computer clusters with many computational and storage resources; it resides at an access point (AP) in the network to provide edge computing services for mobile devices. Mobile applications can enter the network through the AP and ultimately be offloaded to the cloudlets in the network for processing. Compared with that for the traditional central cloud, the spatial distance between cloudlets and mobile devices is closer, which increases the network connection stability and speed between them while reducing the time cost of mobile devices expended on obtaining additional computational resources.

Existing research work has mainly focused on the problem of computation offloading of general tasks under the conditions of fixed cloudlet placement positions [7,8,9,10]. In such studies, cloudlet placement positions are directly given as a part of the network environment, ignoring the impact on computation offloading results from cloudlet placement positions and the data dependence among mobile application components. In fact, the cloudlet placement position in the network is critical to the execution time of computing tasks offloaded from mobile devices and the resource utilization of cloudlets, especially in large-scale wireless metropolitan area networks (WMANs) with thousands of APs. A WMAN provides network services for mobile users in large-scale metropolitan areas. These networks are usually public infrastructure and operated by the local government [11], which brings the following benefits: (1) the metropolitan area covered by the WMAN has a high population density, and cloudlets can provide computing services for more mobile devices to improve the utilization of cloudlets; (2) in view of the large scale of WMANs, service providers can take advantage of economies of scale to reduce the used price of cloudlets, making cloudlet services more easily accepted by the public. However, the correct placement of cloudlets in WMANs is a major challenge. In addition, the correct cloudlet placement position is highly important to improve the user experience of mobile applications. Failure to properly place cloudlets will result in computation offloading that cannot effectively reduce the execution time of mobile applications, which will negatively affect the user experience for mobile applications. This paper focuses on solving the K(≥1) cloudlet placement problem in large-scale WMANs and fully considers the impact of the data transmission time and execution time of sub-tasks included in workflow applications (WAs). The object is to find a cloudlet placement scheme that minimizes the execution time of WAs. The main contributions of this paper are as follows:

• We propose an abstract model of a cloudlet placement system in the WMAN. In this system model, mobile applications are WAs with complex internal dependencies. With the object of minimizing the execution time of WAs, a detailed mathematical analysis and modeling of the *K* cloudlet placement problem in a WMAN is performed.
• The particle encoding and location update mode processes in the traditional particle swarm optimization (PSO) algorithm are improved, and the cloudlet placement strategy based on the PSO algorithm using genetic algorithm (GA) operators with the encoding library updating mode (PGEL) is proposed. By introducing the update operator of the GA and the encoding library update mode in the particle update process, this strategy solves the problems of easily falling into local optima and redundant operations during the particle update process that are encountered in the traditional PSO algorithm.
• To verify the effectiveness and superiority of the proposed strategy, we conducted sufficient simulation experiments. The experimental results show that the proposed strategy not only obtains a near-optimal cloudlet placement scheme in typical WMANs, but also maintains excellent performance when the WMAN changes. This approach also has a major advantage when evaluated against several classic algorithms.

The rest of the paper is organized as follows. In Section 2, we discuss works related to this paper. In Section 3, we describe the system models and define the problem to be studied. In Section 4, we introduce the cloudlet placement strategy based on PGEL. In Section 5, we present our experimental setup, evaluation, and analysis of the results. Finally, Section 6 concludes the paper.

## 2. Related Work

Limited by hardware technology, battery life, and other factors, mobile devices have limited computational resources and often need the help of remote servers to complete computing tasks efficiently. The research on related problems has also attracted the attention of academia and industry [7,12,13]. During the execution of the computing task, it is encapsulated in the virtual machine and offloaded to the remote server for performance [14]. Due to the mature development of cloud service technology, the destination of most computational offloading is the remote cloud [7,15,16]. However, driven by the many advantages of the cloudlet, it has gradually replaced the cloud servers as a new offloading destination in many scenarios. Compared with the cloud server far away from the user, the cloudlet with certain computational resources in the network has a smaller spatial distance from the user, which greatly reduces the transmission delay involved in the task offloading process and effectively improves the user experience of delay-sensitive applications. For example, the Odesa [17] system is designed to support interactive mobile applications. By offloading some application components to the cloudlet instead of the central cloud, the data transmission time and the execution time of computing tasks are reduced, so that the application can meet the strict response time requirements. Taking into account the quality of service (QoS) requirements of mobile users, Hoang et al. [18] proposed a linear planning solution that offloads computing tasks to an appropriate cloudlet to maximize revenue from service providers. In [19], the authors proposed a novel MEC-based mobility-aware offloading model to solve the intra-Cloudlet offloading scheduling issue and inter-Cloudlet load-aware heterogeneous resource allocation issue in terms of considering the offloading execution efficiency, task processing time constraints, and energy efficiency. Chen et al. [20] proposed an innovative framework that uses a distributed decision-making manner and effectively achieves cooperative load balancing among multiple edges based on reinforcement learning in Industrial IoT environments.

Although the study of cloudlets as a computational offloading destination has received much attention, the impact of the cloudlet placement location on the offloading results is often ignored. Some existing studies assume that cloudlets are used in small network environments such as campuses, companies, and factory parks [21,22,23]. In such network environments, the spatial distance between cloudlets and mobile devices is minimal, so the cloudlet placement has minimal impact on network efficiency. However, the cloudlet placement in a WMAN consisting of thousands of APs becomes extremely important and complex. To improve the execution efficiency of mobile applications, it is imperative to optimize the cloudlet placement location. Specifically, many related studies have focused on the cloudlet placement problem in large-scale networks. The authors reassigned the mobile users to complete the cloudlet placement to balance the workload of each cloudlet, thus minimizing the response time of the system. Bhatta et al. [24] formulated the cloudlet placement problem as a multi-objective integer programming model and showed that it is a computationally NP-hard problem. The authors then proposed a bifactor approximate cloudlet placement (ACP) to tackle its intractability. In [25], a dynamic cloudlet placement method based on a clustering algorithm (DCDM-CA) was proposed to solve the problem of deploying mobile cloudlets for mobile applications. After determining the placement location of the cloudlets, the authors also optimized the computational offloading to minimize the system response latency. In [26], the authors proposed an application development method of the Internet of Things (IoT) based on a runtime model. They tried to manage various IoT devices through the architecture based on runtime software for the first time. Guo et al. [27] formulated the edge cloud placement problem as a multi-objective optimization problem to balance the workload between edge clouds and minimize the service communication delay of mobile users. In [28], the authors studied the cloudlet deployment problem to optimize deployment cost and network latency. When the cloudlets have been deployed in the network, the authors proposed a fault-tolerant cloudlet deployment scheme to ensure acceptable QoS. Zhu et al. [29] investigated a joint cloudlet deployment and task offloading problem with the objectives of minimizing energy consumption and the task response delay of users and the number of deployed cloudlets. After formulating this problem as a mixed-integer nonlinear program and proving its NP-completeness, the authors proposed a modified guided population archive whale optimization algorithm to solve it. In [30], the placement problem of edge servers in the Internet of Vehicles (IoV) was studied, and the six-objective edge server deployment optimization model was constructed by simultaneously considering transmission delay, workload balancing, energy consumption, deployment costs, network reliability, and edge server quantity. In [31], the authors utilized particle swarm optimization (PSO) to reallocate the virtual machines (VMs) in overloaded physical machines (PMs) and to consolidate underloaded PMs for energy savings. In [32], the authors utilized PSO to allocate more kinds of resources and to consolidate VMs across multiple cloud data centers. Tseng et al. [33] formulated a multiobjective optimization problem of resource allocation, which considers the CPU and memory utilization of VMs and PMs and the energy consumption of the data center. The authors proposed a multiobjective genetic algorithm (GA) to dynamically forecast the resource utilization and energy consumption. In [34], the authors defined the network-aware VM placement optimization (NAVMPO) problem based on integer linear programming. They proposed the service-oriented physical machine (PM) selection (SOPMS) algorithm and link-aware VM placement (LAVMP) algorithm to solve the above problem. The proposed methods or frameworks in the above research work are oriented toward simple task scenarios. They do not consider the complex dependencies within computing tasks, limiting further division for them. The comparative analysis of the previous work is illustrated in Table 1. In fact, the fine-grained division of computing tasks will effectively improve the utilization efficiency of computational resources.

## 3. System Model and Problem Formulation

In this section, we first describe the WMAN and WA system models. We then define the key time points in the execution process of WAs. Finally, we define the problem precisely. For the ease of reference, we list the key notations of our system model in Table 2.

### 3.1. System Model

#### 3.1.1. WMAN Model

**Figure 1:**
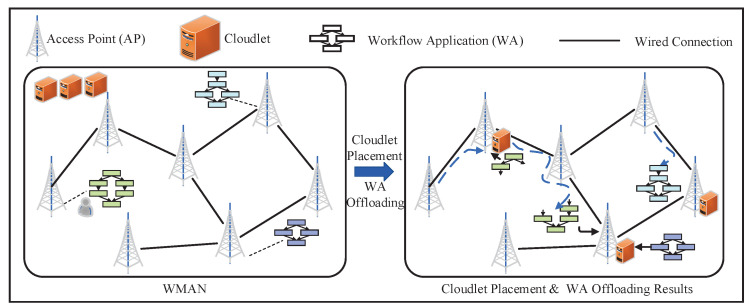
Cloudlet placement and WA offloading in a WMAN.

As shown in Figure 1, we considered a WMAN composed of several APs. The APs are connected through a wired network to form a connectivity graph structure. It is assumed that each AP in the WMAN will receive a WA in the considered time slot, and these WAs are jointly executed by several cloudlets placed on the APs. The cloudlet placement scheme and the offloading strategy of WAs will affect the execution time of WAs. A WMAN can be represented by a graph G=(V,E), and each node in $V=\{v_{1},v_{2},\cdots ,v_{n}\}$ represents an AP in the WMAN. *E* is the set of edges between APs, and each edge $(v_{i},v_{j})\in E$ represents the unit data transmission delay between $v_{i}$ and $v_{j}$. In particular, when two APs are not directly connected, the unit data transmission delay is obtained by adding the unit data transmission delay of each side in the shortest path (in a given AP network topology, the shortest path between APs can be obtained by the Dijkstra algorithm) between them. We define a matrix $D\in R^{n\times n}$, where $D_{i,j}\in D$ represents the unit data transmission delay between $v_{i}$ and $v_{j}$.

#### 3.1.2. WA Model

**Figure 2:**
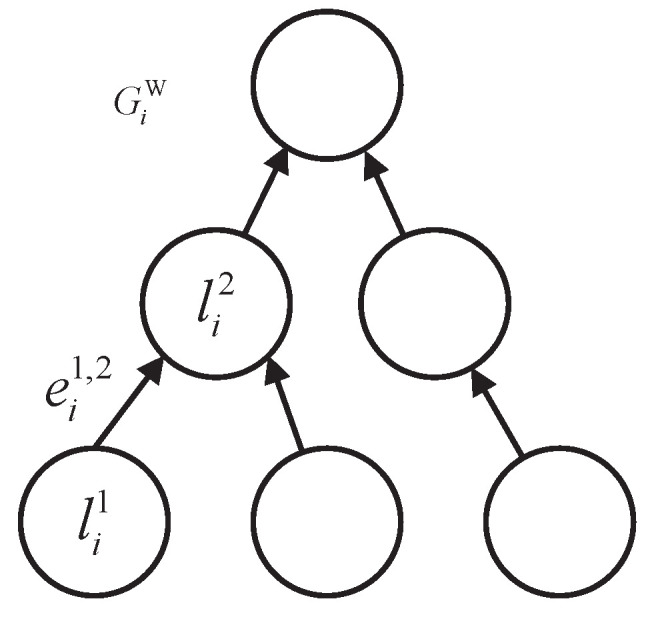
Diagram of the WA.

In a WMAN, the WA received by an AP consists of several sub-tasks, and there is data dependency between them. The set of WAs received by all APs is denoted as $G^{W}=\left\{G_{1}^{W},G_{2}^{W},\cdots ,G_{n}^{W}\right\}$, and each WA can be represented by a directed acyclic graph $G_{i}^{W}=\left(L_{i},E_{i}\right)$. As shown in Figure 2, $L_{i}=\left\{l_{i}^{1},l_{i}^{2},\cdots ,l_{i}^{s}\right\}$ represents *s* sub-tasks included in $G_{i}^{W}$, and the computational requirement of $l_{i}^{j}$ is represented by $\theta_{i}^{j}$. $E_{i}=\left\{e_{i}^{j,k},\forall j,k\in \left\{1,\cdots ,s\right\}\right\}$ indicates the data dependence between sub-tasks included in $G_{i}^{W}$; when $e_{i}^{j,k}>0$, there is a data dependency relationship between $l_{i}^{j}$ and $l_{i}^{k}$, and when $e_{i}^{j,k}=0$, there is no data dependency relationship between $l_{i}^{j}$ and $l_{i}^{k}$.

#### 3.1.3. Cloudlet Placement and Sub-Task Offloading

We use a set $C=\{c_{1},c_{2},\cdots ,c_{K}\}$ to represent *K* cloudlets to be placed in a WMAN, where the computing power of cloudlet $c_{i}$ is represented by $\eta_{i}$. The *K* cloudlet placement scheme in the WMAN can be defined as $\omega =\{\omega_{i,j},\forall i\in \left\{1,\cdots ,K\right\},j\in \left\{1,\cdots ,n\right\}\}$. When cloudlet $c_{i}$ is placed at AP $v_{j}$, $\omega_{i,j}=1$; otherwise, $\omega_{i,j}=0$.

Because our work focuses on cloudlet placement in a WMAN, it does not consider the impact of the sub-task offloading strategy on the WA execution results. When the cloudlet placement positions are determined, we divide the whole WMAN into several areas on average. In each area, a greedy offloading algorithm similar to that in [35] is used to offload the sub-tasks; that is, the WAs received by each area are jointly executed by the cloudlets placed in the area. The execution order of the sub-tasks included in the same WA is determined by the breadth-first traversal (BFT) of the graph [7]. When the above greedy offloading algorithm offloads the sub-tasks, the strategy of minimizing the total time cost of task execution, namely the sum of execution time and transmission time, is adopted. According to the execution location of each sub-task’s precursor sub-tasks, it is offloaded to the appropriate cloudlet, and the global sub-task offloading strategy is ultimately obtained, which is denoted as $M={⋃}_{i=1}^{|G^{W}|}\left\{\left(l_{i}^{j},c_{k}\right)|l_{i}^{j}\in L_{i},c_{k}\in C\right\}$.

#### 3.1.4. WA Execution Time

Given the cloudlet placement scheme ω and sub-task offloading strategy M, the following key time points are defined.

**The start execution time of the sub-task**. The start time of sub-task $l_{i}^{j}$ on cloudlet $c_{k}$ is determined by the execution end time and the dependent data transmission time of all its predecessor sub-tasks. It is quantified as

(1) $$t_{start}\left(l_{i}^{j},c_{k}\right)=\max_{e_{i}^{p,j}>0,\forall e_{i}^{p,j}}\;\left\{t_{end}\left(l_{i}^{p},c_{w}\right)+t_{trs}\left(l_{i}^{p},l_{i}^{j},c_{w},c_{k}\right)\right\},forall\left(l_{i}^{j},c_{k}\right),\left(l_{i}^{p},c_{w}\right)\in M,$$

where $t_{end}\left(l_{i}^{p},c_{w}\right)$ and $t_{trs}\left(l_{i}^{p},l_{i}^{j},c_{w},c_{k}\right)$ are the execution end time of sub-task $l_{i}^{p}$ and the dependent data transmission time between sub-tasks $l_{i}^{p}$ and $l_{i}^{j}$, respectively. Their calculation equations will be given in Equation (4) and Equation (3), respectively.

**The execution time of the sub-task**. The execution time of sub-task $l_{i}^{j}$ is determined by its computational requirements and the computing power of the cloudlet executing the sub-task. It is quantified as

(2) $$t_{exe}\left(l_{i}^{j},c_{k}\right)=\frac{\theta_{i}^{j}}{\eta_{k}},\forall \left(l_{i}^{j},c_{k}\right)\in M.$$

**The dependent data transmission time between the sub-tasks**. The dependent data transmission time between sub-tasks $l_{i}^{p}$ and $l_{i}^{j}$ is determined by the size of the dependent data between the two sub-tasks and the unit data transmission delay between the APs with cloudlets placed. It is quantified as

(3) $$t_{trs}\left(l_{i}^{p},l_{i}^{j},c_{w},c_{k}\right)=e_{i}^{p,j}d_{w^{\prime },k^{\prime }},\forall \left(l_{i}^{j},c_{k}\right),\left(l_{i}^{p},c_{w}\right)\in M,\omega_{w,w^{\prime }},\omega_{k,k^{\prime }}=1.$$

**The execution end time of the sub-task**. The execution end time of sub-task $l_{i}^{j}$ on cloudlet $c_{k}$ is determined by the start execution time of sub-task $l_{i}^{j}$ and its execution time on cloudlet $c_{k}$. It is quantified as

(4) $$t_{end}\left(l_{i}^{j},c_{k}\right)=t_{start}\left(l_{i}^{j},c_{k}\right)+t_{exe}\left(l_{i}^{j},c_{k}\right),\forall \left(l_{i}^{j},c_{k}\right)\in M.$$

**The execution end time of the WA**. The execution end time of WA $G_{i}^{W}$ is the maximum of the execution end time of its sub-tasks. It is quantified as

(5) $$t_{end}\left(G_{i}^{W}\right)=\max_{l_{i}^{j}\in L_{i}}\left\{t_{end}\left(l_{i}^{j},c_{k}\right)\right\},\forall \left(l_{i}^{j},c_{k}\right)\in M.$$

**The execution end time of all WAs**. The end time of all WAs in the WMAN is the maximum of the execution end time of all the WAs. It is quantified as

(6) $$t_{end}\left(G^{W}\right)=\max_{G_{i}^{W}\in G^{W}}\left\{t_{end}\left(G_{i}^{W}\right)\right\}.$$

### 3.2. Problem Formulation

The *K* cloudlet placement based on the WA problem (KCPWP) in a WMAN is defined as follows. Given an integer K>1 and system model parameters $\left(G,D,G^{W}\right)$, the problem is to find ω such that the execution time of WAs in Equation (6) is minimized, i.e.,

(7) $$\begin{array}{ccc} \min_{\omega } & t_{end}\left(G^{W}\right) & \\ s.t. & \sum_{j=1}^{n}\omega_{i,j}=1,\forall i\in \left\{1,\cdots ,K\right\} & \left(C1\right)\\ \; & \sum_{i=1}^{K}\omega_{i,j}\leq 1,\forall j\in \left\{1,\cdots ,n\right\} & \left(C2\right) \end{array}$$

Constraint (C1) indicates that each cloudlet to be placed has only one placement position, and Constraint (C2) indicates that each AP has at most one cloudlet to be placed.

## 4. Cloudlet Placement Strategy Based on PGEL

In this section, we first introduce the traditional PSO algorithm and then introduce the cloudlet placement strategy based on PGEL in detail.

### 4.1. Traditional PSO

The PSO algorithm is a stochastic optimization technique based on populations and was proposed by Eberhart and Kennedy in 1995 [36]. In nature, animals that belong to the same population cooperate with each other in a certain way. Each member of the population changes its behavior by learning its own and others’ experiences. The PSO algorithm solves the optimization problem by imitating the clustering behavior of animals. In the PSO algorithm, a particle represents a candidate solution of the optimization problem, and all particles can move in the whole solution space. In the process of each search, the particle moves at a certain speed, which is affected by three factors: the situation of the particle itself, the best position of the particle itself, and the historical best position of the particle in the whole particle swarm. Although the traditional PSO algorithm has the advantages of good robustness and easy convergence, it also has defects of developing premature convergence and becoming trapped in a local optimum. In this paper, by introducing the crossover operator and mutation operator of the GA into the traditional PSO algorithm, the PGEL cloudlet placement algorithm is proposed to compensate for the defects of the traditional PSO algorithm, optimize its optimization ability, and better solve the *K* cloudlet placement problem in WMANs.

### 4.2. PGEL

#### 4.2.1. Problem Encoding

To solve the KCPWP in WMANs, we use the cloudlet position sequence encoding strategy to encode the particles. Each particle in the particle swarm composed of Ω particles represents a placement scheme of *K* cloudlets in a WMAN. The state of the *i*-th particle in the *t*-th iteration is as follows:

(8) $$P_{i}^{t}=\left(p_{i,1}^{t},p_{i,2}^{t},\cdots ,p_{i,K}^{t}\right),$$

where $p_{i,j}^{t}\in \left\{1,2,\cdots ,n\right\}$ represents the placement position of the *j*-th cloudlet mapped by particle *i* in the *t*-th iteration.

#### 4.2.2. Fitness Function

To judge the cloudlet placement scheme corresponding to each particle, a fitness function is introduced. Our purpose is to obtain a cloudlet placement scheme that can minimize the execution time of WAs in a WMAN. Therefore, the particle with a smaller execution time corresponding to the mapped cloudlet placement scheme can be simply regarded as a better particle. The fitness function of particle $P_{i}^{t}$ can be defined as

(9) $$fitness\left(P_{i}^{t}\right)=Time\left(P_{i}^{t}\right),$$

where $Time(P_{i}^{t})$ represents the execution time of WAs calculated by Equation (6) when the placement scheme corresponding to $P_{i}^{t}$ is adopted. The particle with a smaller fitness obviously corresponds to the cloudlet placement scheme with a shorter execution time for WAs.

#### 4.2.3. Update Strategy

In our previous work [37], the update strategy of the traditional PSO algorithm was introduced in detail. The PSO includes three core parts: inertia, personal cognition, and social cognition. In the iterative process of the algorithm, the update of each particle is affected by its personal optimal position and the current global optimal position. To avoid the tendency of the traditional PSO algorithm falling into the local optimum prematurely and to enhance the search ability of the algorithm, we introduce the crossover operator in the personal cognitive and social cognitive domains and the mutation operator in the inertia part to compensate for the defects of the traditional PSO algorithm. The update strategy of particle *i* in the (t+1)-th iteration is as follows:

(10) $$P_{i}^{t+1}=SC\left(PC\left(IT\left(P_{i}^{t},w^{t+1},\mu \right),pBest_{i}^{t},c_{1}^{t+1}\right),gBest^{t},c_{2}^{t+1}\right),$$

where PC(x,y,z) and SC(x,y,z) are the personal cognition update operation and the social cognition update operation, respectively, and IT(x,y) is the inertia update operation.

**Figure 3:**
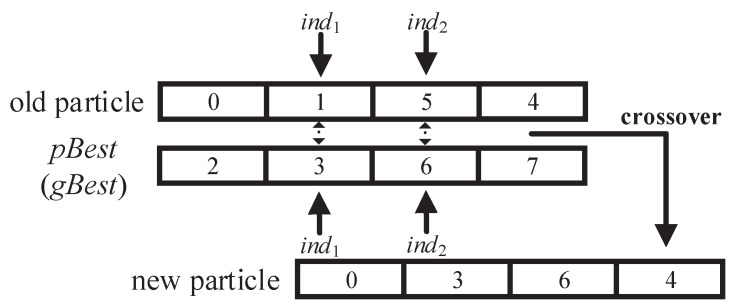
Crossover operator in the personal cognitive update operation and the social cognitive update operation.

The crossover operator of the GA is introduced into the personal cognitive update operation and social cognitive update operation. The results of the personal cognition update operation and social cognition update operation are quantified as

(11) $$PC\left(A_{i}^{t+1},pBest_{i}^{t},c_{1}^{t+1}\right)=\left\{\begin{array}{cc} CO(A_{i}^{t+1},pBest_{i}^{t}) & ,\;\mathrm{if}\;r_{1}\leq c_{1}^{t+1}\\ A_{i}^{t+1} & ,\;\mathrm{if}\;r_{1}>c_{1}^{t+1} \end{array}\right.,$$

(12) $$SC\left(B_{i}^{t+1},gBest^{t},c_{2}^{t+1}\right)=\left\{\begin{array}{cc} CO(B_{i}^{t+1},gBest^{t}) & ,\;\mathrm{if}\;r_{2}\leq c_{2}^{t+1}\\ B_{i}^{t+1} & ,\;\mathrm{if}\;r_{2}>c_{2}^{t+1} \end{array}\right.,$$

respectively, where $r_{1}$ and $r_{2}$ are random numbers from 0 to 1 and CO(x,y) represents the crossover operator of GA. The crossover operator randomly selects an encoded segment of particle *x* to be updated and replaces it with the corresponding encoded segment of particle *y*. The crossover operator in the personal (or social) cognitive update operation is shown in Figure 3.

**Figure 4:**
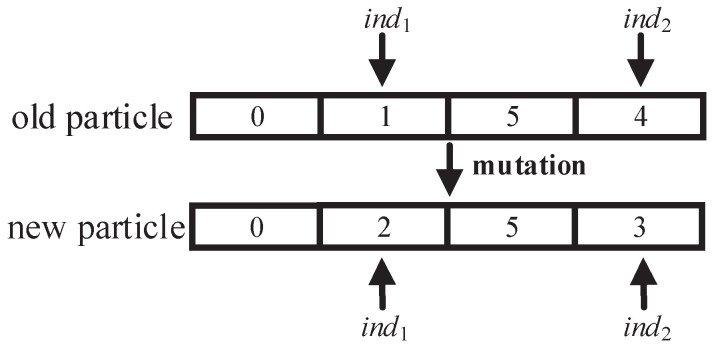
Mutation operator in the inertial update operation.

The mutation operator of the GA is introduced into the inertia update operation. The result of the inertia update operation is as follows:

(13) $$IT\left(P_{i}^{t},w^{t+1},\mu \right)=\left\{\begin{array}{cc} MU(P_{i}^{t},\mu ) & ,\;\mathrm{if}\;r_{3}\leq w^{t+1}\\ P_{i}^{t} & ,\;\mathrm{if}\;r_{3}>w^{t+1} \end{array}\right.,$$

where $r_{3}$ is a random number from 0 to 1 and MU(x) represents the mutation operator of the GA. The mutation operator randomly selects μ encodings of particle *x* to be updated and then randomly changes the values of these encodings. The mutation operator in the inertial update operation is shown in Figure 4. When μ=2, the encoded $ind_{1}$ and $ind_{2}$ are selected; that is, the placement positions of the corresponding two cloudlets have changed.

*A.*  Traditional updating mode.

**Figure 5:**
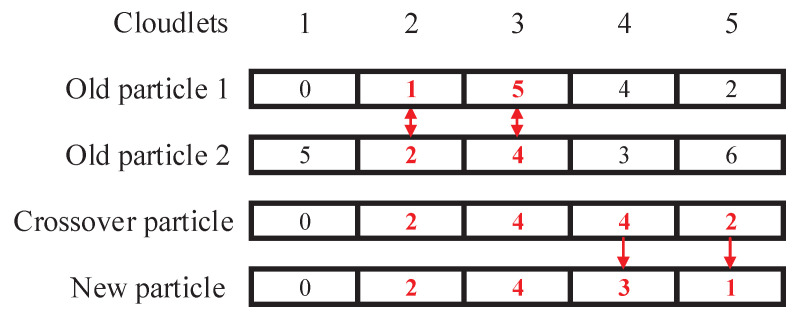
Traditional crossover operator. (The red parts indicate the crossover and adjustment, and the arrows indicate the crossover and adjustment operator.)

**Figure 6:**
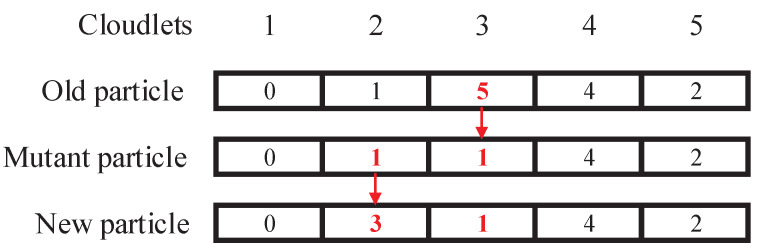
Traditional mutation operator. (The red parts indicate the mutation and adjustment, and the arrows indicate the mutation and adjustment operator.)

In our previous work [37], we used the traditional updating mode to update the encodings of the particle. The traditional updating mode is as follows:

(1) Initialization: Randomly encode all encoding bits of the particle and ensure that the encodings are not equal.
(2) Crossover: If the particle’s encoding obtained by the crossover operator conflicts with the original encoding, it does not meet the constraint that an AP places only a cloudlet, but needs to adjust the encoding according to certain rules (such as the most equivalent replacement method) to ensure that every two encodings are not equal. As shown in Figure 5, the crossed 2 and 4 conflict with the original encoding and need to be adjusted to avoid conflict.
(3) Mutation: Similarly, if the particle’s encoding obtained by the mutation operator conflicts with the original encoding, the mutated encoding needs to be adjusted to ensure that the two encodings are not equal. As shown in Figure 6, 5 in the original encoding changes to 1, and the new encoding conflicts with the original encoding. One of the adjustment schemes is to change 1 to 5 in the mutated encoding.

After the mutation operator and crossover operator are executed in the traditional updating mode, the encodings in the same particle may conflict and need an additional adjustment process, which will result in additional execution time during the execution process of the algorithm.

*B.*  Encoding library updating mode.

In PGEL, we use the encoding library updating mode to avoid possible encoding conflicts to avoid the extra time caused by the adjustment process. We give the encoding library for each particle and update it in the following way:

(1) Initialization: When the particles are initialized, the encodings are removed from the encoding library one by one so that they do not conflict.
(2) Crossover: In the process of executing the crossover operator, first add the replaced encodings of the particle back to their own encoding library, and then, check the encodings obtained by the cross one by one. If encoding exists in the encoding library, delete it from the encoding library. If encoding does not exist in the encoding library, the closest encoding is selected from the encoding library to replace it. The label of the AP is determined by its specific spatial location, so the location of the access point in the network represented by the closest value is the closest, so the above operation can retain the original particle placement scheme to the greatest extent.
(3) Mutation: When the mutation operator needs to be executed, an encoding is randomly selected from the encoding library to replace the original encoding, and then, the original encoding is added to the encoding library.

The encoding library update mode can ensure that the encodings in the particles do not conflict and effectively shorten the execution time of the crossover operator and the mutation operator.

#### 4.2.4. Map from a Particle-to-Cloudlet Placement Scheme

The mapping algorithm of the particle-to-cloudlet placement scheme is shown in Algorithm 1.   
**Algorithm 1:** Mapping particle to cloudlet placement scheme
   **Input**: Paticle $P_{i}^{t}$.
   **Output**: Cloudlet placement scheme ω.**1** **begin****2**  **foreach** 
$\omega_{i,j}\in \omega$ **do****3**   
$\omega_{i,j}=0$;**4**  **end****5**  **foreach** 
$p_{i,j}^{t}\in P_{i}^{t}$ **do****6**   
$\omega_{j,p_{i,j}^{t}}=1$;**7**  **end****8**  **return** 
ω**9** 
**end**


The input of Algorithm 1 is particle $P_{i}^{t}$, and the output is cloudlet placement scheme ω. First, cloudlet placement scheme ω is initialized (Lines 2–4); second, the placement position of each cloudlet is determined according to each encoding of the particle (Lines 5–7); lastly, cloudlet placement scheme ω is output (Line 8).

#### 4.2.5. Parameter Settings

The inertia weight factor *w* determines the convergence and search ability of PGEL, so its setting is very important. According to Equation (13), when *w* is small, the probability of particle mutation is small, and the current state of the particle has a great influence on the update of the next state, so PGEL has a strong local search ability. In contrast, when *w* is large, the probability of particle mutation is large, and the current state of particle has little influence on the next state update, so PGEL has a strong global search ability. In the early stage of algorithm implementation, our emphasis is on giving more attention to the global search ability. As the search deepens, our focus is on concentrating more attention on the local search ability. In conclusion, *w* should evolve with the evolution of population particles. In PGEL, an inertia weight factor adjustment strategy is used, which is adjusted adaptively according to the advantages and disadvantages of the current population of particles. In the *t*-th iteration, the value of the inertia weight factor is as follows:

(14) $$w^{t}=w_{\max}-\left(w_{\max}-w_{\min}\right)\times \exp\left(\frac{d(P^{t-1})}{d(P^{t-1})-1.01n}\right),$$

where $w_{\max}$ and $w_{\min}$ are the maximum and minimum values of the inertia weight factor, respectively, and $d(P^{t-1})$ is the number of bits encoded differently between particle $P^{t-1}$ and the global best particle.

In addition, the personal cognitive factor $c_{1}^{\max}$ and the social cognitive factor $c_{2}^{\max}$ are set by the linearly increasing strategy and the linearly decreasing strategy in the iterative process [38], respectively. They are quantified as

(15) $$c_{1}^{t}=c_{1}^{\min}+t\times \frac{c_{1}^{\max}-c_{1}^{\min}}{t_{\max}},$$

(16) $$c_{2}^{t}=c_{2}^{\max}-t\times \frac{c_{2}^{\max}-c_{2}^{\min}}{t_{\max}},$$

respectively, where $c_{1}^{\max}$ and $c_{2}^{\max}$ are the maximum values of the personal cognitive factor and social cognitive factor, respectively; $c_{1}^{\min}$ and $c_{2}^{\min}$ are the minimum values of the personal cognitive factor and social cognitive factor, respectively; and $t_{\max}$ is the maximum number of iterations of the algorithm.

#### 4.2.6. Algorithm Flow

The main steps of PGEL are as follows:

Step 1: The parameters of PGEL are initialized, and then, the initial population is generated.

Step 2: According to the mapping between cloudlets and APs, the fitness of each particle is calculated according to Equation (9). The personal best state of each particle is initialized by its initial state, and the particle with the least fitness in the initial particle swarm is set as the global best particle.

Step 3: The particles are updated one by one according to Equation (10), and the fitness of the particles is calculated after updating.

Step 4: If the fitness of the updated particle is less than that of its personal best, it is set as its personal best. Otherwise, go to Step 6.

Step 5: If the fitness of the updated particle is less than that of the global best particle, it is set as the global best particle.

Step 6: Verify that the stop condition is met. If the stop condition is not satisfied, return to Step 3; otherwise, the algorithm will terminate.

### 4.3. Time Complexity

In each iteration of PGEL, all particles need to be updated and recalculate the corresponding fitness. In an iteration, the number of particle updates is jointly determined by the population size Ω and particle dimension Φ. The time complexity of the fitness value calculation is determined by the number of cloudlets to be placed (*K*). Since the particle dimension is equal to the number of cloudlets, that is Φ=K, the time complexity of each iteration of PGEL is O(Ω·K).

## 5. Performance Evaluation

In this section, to verify the effectiveness of the proposed placement strategy based on PGEL in solving the KCPWP in WMANs, the experimental evaluation was carried out in a simulation environment. In particular, the following research questions (RQs) were verified by simulation experiments:

• RQ1: Can PGEL obtain a near-optimal cloudlet placement scheme in typical WMANs? (Section 5.3)
• RQ2: What is the impact of changes in the WMAN on the performance of PGEL? (Section 5.4)
• RQ3: In typical WMANs, compared with several classic algorithms, does PGEL have performance advantages when solving KCPWP? (Section 5.5)

For RQ1, the experimental results show that PGEL can obtain a near-optimal cloudlet placement scheme in typical WMANs. For RQ2, it can be seen from the experimental results that regardless of how WMANs change, the performance of PGEL is not affected, and a near-optimal cloudlet placement scheme can be obtained. For RQ3, the experimental results show that PGEL is superior to other algorithms in typical WMANs.

### 5.1. Experimental Settings

All simulation experiments in this section were carried out on a PC equipped with an i5-8500 CPU and 32 GB of RAM. The operating system version was Windows 10-2004. PGEL and all classic algorithms were implemented in Python 3.7. The relevant parameters of PGEL refer to [37] and were set as $t_{\max}=1000$, Ω=100, $w_{\max}=0.9$, $w_{\max}=0.4$, $c_{1}^{\max}=0.9$, $c_{1}^{\min}=0.2$, $c_{2}^{\max}=0.9$, $c_{2}^{\min}=0.4$, and μ=0.1K.

We selected the regions from the dataset of the Shanghai Telecom base station [39] to simulate the WMANs according to a certain longitude and latitude span. Different WMANs include different numbers and connection topologies for APs. The unit data transmission delay of each edge in the WMAN was generated randomly from 5 ms to 50 ms [4]. During the process of sub-task offloading, the WMAN was divided into 2 × 2 areas.

### 5.2. Classic Algorithms

To verify the advantages of PGEL, we introduced the following classic algorithms in the simulation experiments:

• Optimal placement algorithm (OPT): This algorithm traverses all possible cloudlet placement schemes and selects the placement scheme with the minimum execution time of WAs as the optimal cloudlet placement scheme.
• PSO: This traditional PSO algorithm has the same encoding and parameter settings as PGEL.
• GA: According to the update strategy of the GA, it uses the elite retention strategy, binary tournament selection operator, two-point crossover operator, and exchange mutation operator to update the chromosome and takes the final elite solution as the optimal solution. The crossover and mutation probability were set as 0.7 and 0.1, respectively.
• Random cloudlet placement algorithm (RAN): This algorithm randomly selects *K* from all APs of the WMAN and randomly places *K* to-be-placed cloudlets on these APs. The result is an average of 100 repetitions.

### 5.3. RQ1. PGEL Can Obtain a Near-Optimal Cloudlet Placement Scheme in Typical WMANs

According to the administrative divisions of Shanghai, we selected regions with spans of 8 and 4 points in latitude and longitude, respectively, from Huangpu, Xuhui, Minhang, and Pudong as typical WMANs. The distribution and topology of APs in the WMAN were simulated by the data of the Telecom base station in [39]. It was assumed that the number of to-be-placed cloudlets *K* in the WMAN is determined by the number of APs *n*; that is, K=κ·n,κ∈(0,1). According to the population density of the above administrative divisions, the κ values of the selected regions from Huangpu, Xuhui, Minhang, and Pudong were set to be 0.35, 0.3, 0.25, and 0.2, respectively. In the above regions, the WAs received by APs were randomly generated from various network architectures, namely AlexNet, Visual Geometry Group network (VGGNet), GoogLeNet, and residual network (ResNet) [40]. In addition, the clock frequency of the CPU equipped with the to-be-placed cloudlets in the same WMAN satisfied a uniform distribution of 2 GHz to 3 GHz.

**Figure 7:**
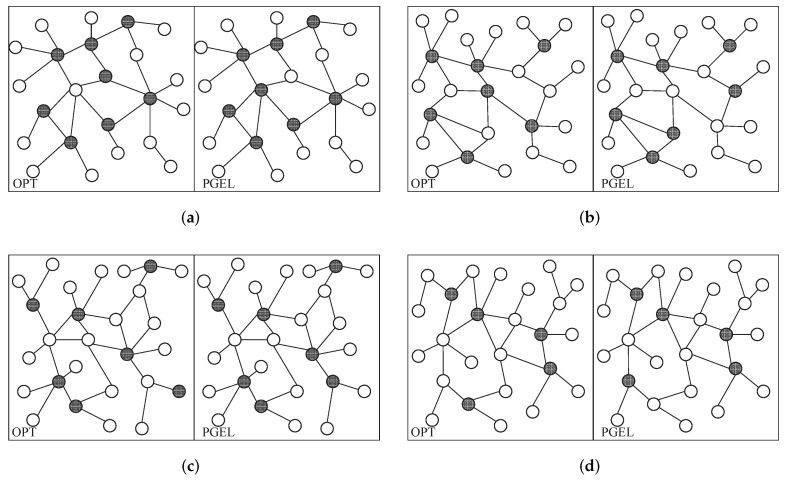
Comparison of local cloudlet placement results in typical WMANs. (**a**) Huangpu. (**b**) Xuhui. (**c**) Minhang. (**d**) Pudong.

By running OPT and PGEL in different typical WMANs, we can obtain the local cloudlet placement results, which are shown in Figure 7. Although the cloudlet placement scheme obtained by PGEL in each typical WMAN is not equivalent to the optimal cloudlet placement scheme, there are only 1-2 cloudlet placement positions that differ locally; other cloudlet placement positions are the same, and the overall placement scheme is close.

**Figure 8:**
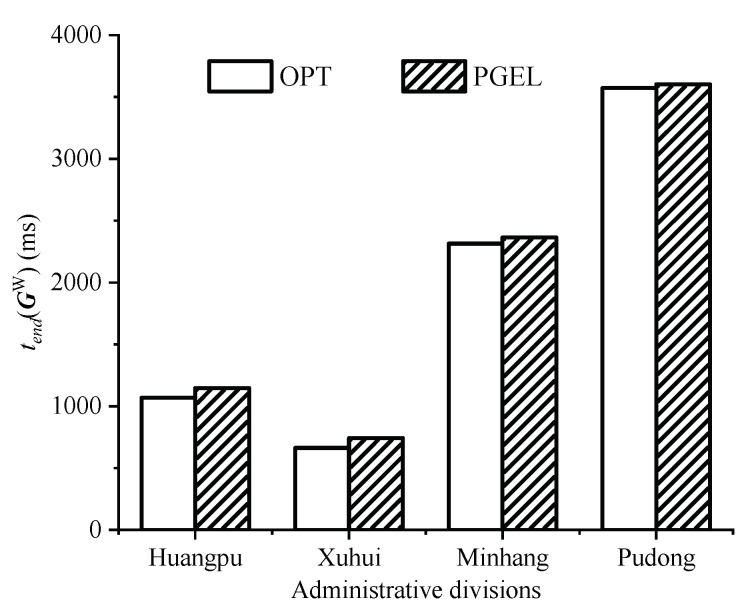
Comparison of execution times of WAs in typical WMANs.

By calculating the execution time of the WAs corresponding to the cloudlet placement scheme shown in Figure 7, the histogram shown in Figure 8 can be obtained. The results show that in four different typical WMANs (Huangpu, Xuhui, Minhang, and Pudong), and the execution times of the WAs corresponding to the cloudlet placement scheme obtained by PGEL were 7.51%, 11.91%, 2.06%, and 0.83% larger than those corresponding to the optimal placement scheme, respectively. The combined insights of Figure 7 and Figure 8 indicate that although the cloudlet placement schemes obtained by PGEL in the WMANs somewhat differed from the optimal placement schemes, the difference was very small. PGEL can obtain the approximate optimal solution of KCPWP in typical WMANs. Although there was still a certain gap between the optimal placement scheme and the placement scheme obtained by PGEL, it is unfeasible to obtain the optimal placement scheme by brute force when the network scale increases, so PGEL has a certain practicality.

### 5.4. RQ2. The Changes in the WMAN Have Almost No Impact on the Performance of PGEL

From Section 5.3, we know that PGEL can very closely approach the optimal cloudlet placement scheme in typical WMANs. However, the WMAN cannot be fixed; it is both temporal and spatial. Next, we will discuss the impact of WMAN changes on PGEL performance.

#### 5.4.1. The Impact of Changes in AP Topology on the Performance of PGEL

We used the regions selected from different administrative divisions of Shanghai as described in Section 5.3 to simulate changes in AP topology. In this part, it is assumed that the number of to-be-placed cloudlets in each region and the clock frequency of the CPU equipped with the to-be-placed cloudlets are the same, and other settings were the same as those in Section 5.3. In addition, the WAs received by APs were simulated by GoogLeNet, the structure of which is shown in [41].

**Figure 9:**
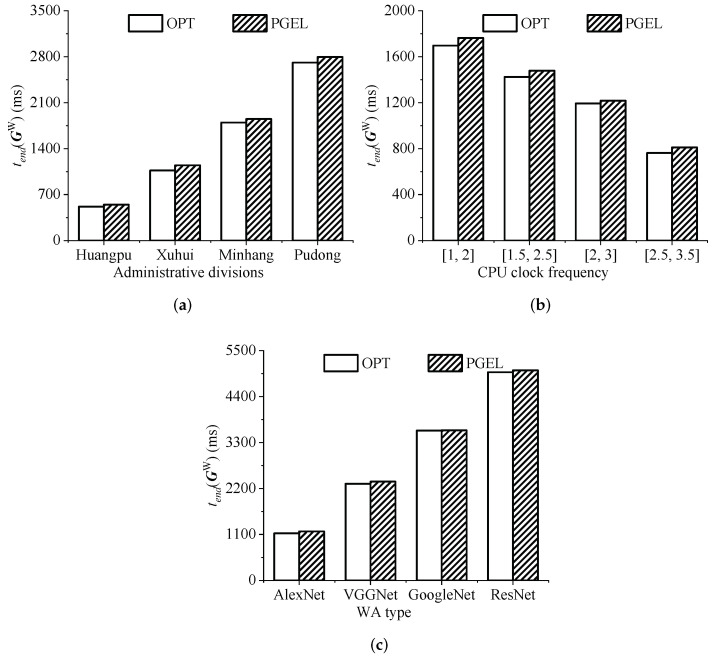
The impact of WMAN changes on the performance of PGEL. (**a**) APs’ topology changes. (**b**) CPU clock frequency changes. (**c**) WA changes.

As shown in Figure 9a, in four WMANs with different AP topologies, although the number of to-be-placed cloudlets and their CPU clock frequency were the same, the execution times of the identical number and type of WAs were different. This is because the AP topology will affect the placement positions of cloudlets, resulting in different sub-task offloading schemes and data transmission times. From the experimental results, it can be concluded that in four WMANs with different AP topologies (Huangpu, Xuhui, Minhang, and Pudong), the executions time of the WAs corresponding to the cloudlet placement scheme obtained by PGEL were 5.80%, 7.51%, 3.04%, and 3.13% larger than those corresponding to the optimal placement scheme, respectively. Although there was a gap between them, it can almost be ignored. On the basis of these results, we can conclude that the changes in the AP topology have little impact on the performance of PGEL, and PGEL can still obtain a near-optimal cloudlet placement scheme.

#### 5.4.2. The Impact of the Changes in the To-Be-Placed Cloudlet CPU Clock Frequency on the Performance of PGEL

In Section 5.3, we assume that the CPU clock frequency of to-be-placed cloudlets meets the uniform distribution within a certain range. In this part, we simulated the changes in the CPU clock frequency of the to-be-placed cloudlets by changing the maximum and minimum values of the uniform distribution. In addition, the first typical WMAN (i.e., Huangpu) of Section 5.3 was adopted. The WAs received by APs were simulated by implementing GoogLeNet.

The results shown in Figure 9b indicate that the execution time of the WAs was negatively correlated with the CPU clock frequency of to-be-placed cloudlets. This is because the CPU clock frequency determines the execution time of sub-tasks. The greater the CPU clock frequency is, the smaller the execution time of sub-tasks. The execution time of sub-tasks largely determines the execution time of WAs. In addition, the experimental results indicate that the execution time of the WAs corresponding to the cloud placement scheme obtained by PGEL was 3.94%, 3.76%, 2.03%, and 6.41% larger than the optimal placement scheme under the four to-be-placed cloudlet CPU clock frequencies, respectively. Although there was a gap between the values, it can almost be ignored. From these results, we can conclude that the CPU clock frequency changes of the to-be-placed cloudlets have little impact on the performance of PGEL, and PGEL can still be used to obtain the near-optimal cloudlet placement scheme.

#### 5.4.3. The Impact of the Changes in WAs on the Performance of PGEL

In Section 5.3, we assume that the WAs randomly select from four typical tasks. In this part, we simulated the changes in WAs by changing the type of WAs. In addition, the relevant settings of the first typical WMAN of Section 5.3 were adopted, and all WAs were equivalent.

As shown in Figure 9c, when the types of WAs were different, the execution times of the WAs were different. This is because different types of WAs have different computational requirements and data transmission rates, so the execution time was different. From the experimental results, when the WA types were AlexNet, VGGNet, GoogLeNet, and ResNet, the execution times of the WAs corresponding to the cloudlet placement scheme obtained by PGEL were 4.27%, 2.06%, 0.15%, and 0.95% larger, respectively, than those corresponding to the optimal placement scheme. Although there was a gap between the values, it can almost be ignored. From these results, we can conclude that the changes in the WAs have little impact on the performance of PGEL, and PGEL can still obtain a near-optimal cloudlet placement scheme.

In summary, we can conclude that changes in the WMANs have almost no impact on the performance of PGEL, and PGEL can still obtain a near-optimal cloudlet placement scheme.

### 5.5. RQ3. PGEL Has Greater Performance Advantages than Several Classic Algorithms in Typical WMANs Do

**Figure 10:**
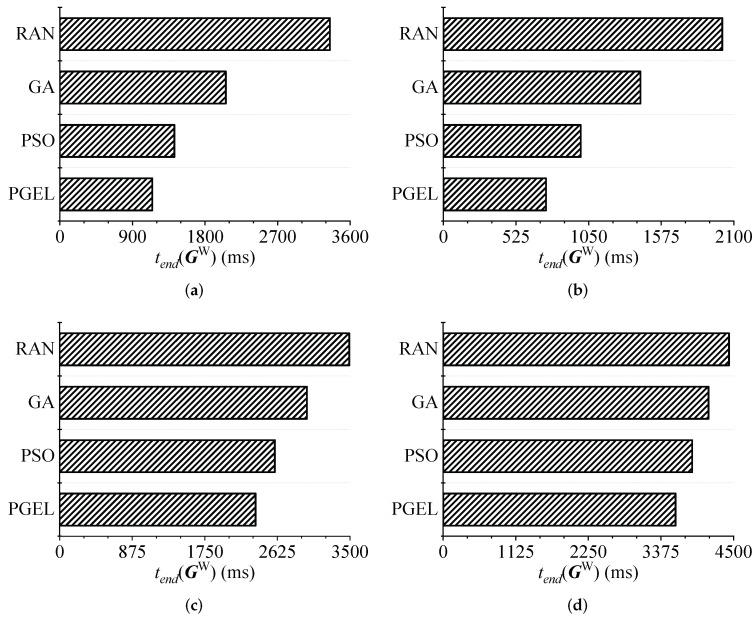
Comparison of cloudlet placement. (**a**) Huangpu. (**b**) Xuhui. (**c**) Minhang. (**d**) Pudong.

Finally, we used experiments to test the performance advantages of the proposed PGEL over several classic algorithms when solving the KCPWP in typical WMANs. As shown in Figure 10, in the four typical WMANs discussed in Section 5.3, PGEL reduced the execution time of WAs by 15.04%, 31.32%, and 44.99% on average compared with those of the PSO, GA, and RAN, respectively. PGEL adaptively adjusts the search capability according to the current situation and iteratively updates from a global perspective. In the four different WMANs, PGEL performed better than several classic algorithms did. Although the PSO algorithm has a certain search ability, it easily falls into a local optimum, so its performance is limited. The GA performs only a partial search during each iteration, so its performance is poor, and RAN can reflect only the average level of all placement schemes. In summary, it can be concluded that PGEL has a greater performance advantage than do several classic algorithms in solving the KCPWP in WMANs.

## 6. Conclusions

For mobile users seeking solutions to the problem of insufficient resources through remote servers, cloudlet technology is essential. However, the problem of cloudlet placement has been largely ignored. To solve the problem of cloudlet placement, this paper proposed a time-driven cloudlet placement strategy based on PGEL, which aims to reduce the execution time of WAs in WMANs. We set up a reasonable simulation environment and conducted a complete simulation experiment based on this approach. The results show that compared with the existing solutions, our proposed strategy has better performance and is extraordinarily close to the optimal placement scheme.

In the future, we will consider the impact of environmental fluctuations (i.e., network delays, bandwidth fluctuations, and cloudlet failures) on cloudlet placement and improve existing strategies to adapt to the dynamic environment. In addition, we will fully consider the characteristics of the sub-tasks included in the WA and the placement cost of the cloudlet in different positions and design a more scientific and complete system cost model.

## Figures and Tables

**Table 1:** The comparative analysis of different work (“+”: involved; “−”: not involved).

Reference	Infrastructure			Server		Task Type		Constraint		Object			
	Cloud	Edge	Terminal	Single	Multiple	Jobs	Workflow	Energy	Deadline	Time	Energy	Workload	Other
Our work	+	+	+	−	+	−	+	−	+	+	−	−	−
[24]	−	+	+	−	+	+	−	−	−	+	−	−	−
[25]	−	+	+	−	+	+	−	−	−	−	−	−	+
[27]	+	+	+	−	+	+	−	−	−	+	−	+	−
[28]	−	+	+	−	+	+	−	−	+	+	−	−	+
[29]	−	+	+	−	+	+	−	−	−	+	+	−	−
[30]	−	+	+	+	−	+	−	−	−	−	−	−	+
[31]	+	−	+	−	+	+	−	−	−	+	+	−	−
[34]	+	−	+	−	+	+	−	+	−	+	+	−	+

**Table 2:** Summary of key notations.

Notation	Description
G=(V,E)	a WMAN
$v_{i}\in V$	AP with index *i* in the WMAN
$D_{i,j}\in D$	unit data transmission delay between $v_{i}$ and $v_{j}$
$G^{W}=\left\{G_{1}^{W},\cdots ,G_{n}^{W}\right\}$	set of WAs received by all APs
$G_{i}^{W}=\left(L_{i},E_{i}\right)$	WA with index *i*
$L_{i}=\left\{l_{i}^{1},\cdots ,l_{i}^{s}\right\}$	sub-tasks included in WA $G_{i}^{W}$
$E_{i}=\left\{e_{i}^{j,k}\right\}$	data dependence between sub-tasks included in WA $G_{i}^{W}$
$\theta_{i}^{j}$	computational requirement of sub-task $l_{i}^{j}$
$C=\{c_{1},\cdots ,c_{K}\}$	set of cloudlets to be placed in a WMAN
$\eta_{i}$	computing power of cloudlet $c_{i}$
$\omega =\{\omega_{i,j}\}$	cloudlet placement scheme
$M={⋃}_{i=1}^{|G^{W}|}\left\{\left(l_{i}^{j},c_{k}\right)\right\}$	global sub-task offloading strategy
$t_{start}\left(l_{i}^{j},c_{k}\right)$	start time of sub-task $l_{i}^{j}$ on cloudlet $c_{k}$
$t_{exe}\left(l_{i}^{j},c_{k}\right)$	execution time of sub-task $l_{i}^{j}$
$t_{trs}\left(l_{i}^{p},l_{i}^{j},c_{w},c_{k}\right)$	dependent data transmission time between sub-tasks $l_{i}^{p}$ and $l_{i}^{j}$
$t_{end}\left(l_{i}^{p},l_{i}^{j},c_{w},c_{k}\right)$	execution end time of sub-task $l_{i}^{j}$ on cloudlet $c_{k}$
$t_{end}\left(G_{i}^{W}\right)$	execution end time of WA $G_{i}^{W}$
$t_{end}\left(G^{W}\right)$	end time of all WAs in the WMAN

## Data Availability

Data in this paper are available from the corresponding authors upon request.
